# A Phase I Study of Hydroxychloroquine and Suba-Itraconazole in Men with Biochemical Relapse of Prostate Cancer (HITMAN-PC): Dose Escalation Results

**DOI:** 10.1158/2767-9764.CRC-26-0010

**Published:** 2026-03-27

**Authors:** Barak Talmor, Stefano Marastoni, Brandon Lau, Andrew O. Yam, Nicole Yeung, Hui-Ming Lin, Zhu Juan Li, Henry Woo, Ganes Pranavan, Phillip D. Stricker, Lisa G. Horvath, Marianne Koritzinsky, Bradly G. Wouters, Megan Crumbaker, Anthony M. Joshua

**Affiliations:** 1 Kinghorn Cancer Centre, St Vincents Hospital, Sydney, Australia.; 2 Princess Margaret Cancer Centre, Toronto, Canada.; 3 Fiona Stanley Hospital, Perth, Australia.; 4 Garvan Institute of Medical Research, Sydney, Australia.; 5 School of Clinical Medicine, St Vincent’s Healthcare Clinical Campus, Faculty of Medicine and Health, UNSW Sydney, Sydney, Australia.; 6 Chris O’Brien Lifehouse, Camperdown, Australia.; 7 Department of Urology, Blacktown Hospital, Blacktown, Australia.; 8 Blacktown Mount Druitt Clinical School, Western Sydney University, Blacktown, Australia.; 9 Canberra Health Services, Canberra, Australia.; 10 St. Vincent’s Prostate Cancer Research Centre, Sydney, Australia.; 11 University of Sydney, Sydney, Australia.

## Abstract

**Purpose:**

Biochemical recurrence (BCR) of prostate cancer presents a clinical challenge with limited systemic treatment options beyond androgen deprivation therapy (ADT), which carries significant morbidity. Preclinical data suggest that lysosomal homeostasis, including cholesterol trafficking and pH regulation, is a therapeutic vulnerability in hormone-dependent cancers. We therefore conducted HITMAN-PC, a phase I trial evaluating suba-itraconazole (SI) and hydroxychloroquine (HCQ) in men with BCR.

**Patients and Methods:**

The synergy of SI and HCQ was validated in hormone-sensitive and castration-resistant prostate cancer cell lines. The HITMAN-PC trial (NCT03513211) then used a rolling-six design to establish the maximum tolerated dose (MTD) and recommended phase II dose of HCQ with a fixed SI dosage (150 mg twice daily). Secondary endpoints included safety, PSA kinetics [PSA progression-free survival (PFS) and time to ADT], and exploratory pharmacokinetic and lipidomic profiling.

**Results:**

Itraconazole showed dose-dependent cytotoxicity, with synergy in LNCaP-derived hormone-sensitive and -resistant lines. Eleven patients were enrolled. Two dose-limiting toxicities at HCQ 600 mg twice daily (grade 3 diarrhea and alanine aminotransferase elevation) defined this level as the MTD with SI 150 mg twice daily. Common adverse events were hypertension, QTc prolongation, diarrhea, and nausea; no grade 4 events occurred. No PSA declines ≥50% were observed although most patients achieved PSA stabilization. The median PSA-PFS, time to ADT, and metastasis-free survival were 5.5, 14.3, and 15.9 months, respectively. Lipidomic profiling revealed 240 treatment-associated lipid changes, with sphingomyelin and triacylglycerol species correlating with PSA-PFS.

**Conclusions:**

Despite limited clinical activity overall, the identified lipidomic signatures provide proof of concept for using plasma lipidomics to monitor pharmacodynamic activity in future metabolism-targeted trials.

**Significance:**

This study established a safe dose for HCQ and SI but found limited clinical efficacy in prostate cancer. Its significance lies in providing proof of biology that this combination alters systemic lipid metabolism. We identified specific plasma lipid species that correlate with clinical outcomes, highlighting the power of embedded biomarker analysis to generate valuable mechanistic insights and candidate biomarkers, even in the absence of a strong clinical response.

## Introduction

Globally, prostate cancer imposes a substantial health burden, with more than 1.4 million new diagnoses in 2020 alone, ranking it as the second leading cancer worldwide (1). Although the majority of initial diagnosis often reveals localized disease (2), which is amenable to curative radical prostatectomy (RP) or radiotherapy (RT), long-term success is frequently challenged by biochemical recurrence (BCR), which emerges within 10 years in 20% to 40% of men following RP and 30% to 50% after RT treatment (3). A proportion of men with BCR will ultimately progress to metastatic disease, with metastasis-free survival (MFS) estimated at ∼91% at 5 years and ∼77% at 10 years after recurrence. The risk is strongly influenced by PSA kinetics: men with a PSA doubling time (PSADT) ≤10 months develop metastases after a median of ∼16 years, which shortens to ∼12 years when the doubling time is ≤6 months (4, 5). Although androgen deprivation therapy (ADT), or more recently enzalutamide, may prolong MFS (6), it comes at a cost of significant morbidity (7). Thus, there is an unmet need to find treatments that delay the need for ADT while maintaining quality of life in men with BCR prostate cancer (8–11).

Drug repurposing has emerged as a promising strategy to expand the therapeutic arsenal against cancer (12). Among these, itraconazole (ITRA), a widely used antifungal agent, has been investigated across multiple cancer types for its potential anticancer properties (13), including those critical for prostate cancer progression, such as key oncogenic pathways, including angiogenesis, Hedgehog, Wnt/β-catenin, and mTOR signaling (14). Suba-itraconazole (SI), a highly bioavailable formulation of ITRA, may augment the drug’s broad anticancer activity (15, 16).

Clinical trials evaluating ITRA as a monotherapy or in combination with other agents have demonstrated potential benefits across a range of malignancies, including prostate cancer (14). In BCR, ITRA offers a promising noncastrating alternative to delay ADT. Prior studies have shown that it can exert a biological effect without affecting testosterone levels, with nearly half of patients (47%) demonstrating some PSA decline although deep PSA responses (≥50% decline) were infrequent (5%; ref. 17). In metastatic castration-resistant prostate cancer, ITRA resulted in a confirmed PSA response (≥50% decline) in 14% of patients and a median PSA progression-free survival (PFS) of approximately 3.9 months. These effects occurred without lowering testosterone although toxicities consistent with apparent mineralocorticoid excess were common (18).

A key cellular process implicated in prostate cancer survival and therapeutic resistance is lysosome homeostasis (19). This can be targeted by lysosomotropic agents such as hydroxychloroquine (HCQ) and the related compound chloroquine (CQ). These agents accumulate in acidic cellular compartments, raising lysosomal pH, which in turn inhibits hydrolase activity and induces broad lysosomal dysfunction and downstream autophagy inhibition (20). In the context of prostate cancer, this disruption of lysosome homeostasis and downstream processes, such as autophagy, holds therapeutic potential for enhancing the efficacy of other treatments such as docetaxel (21) and BH3 mimetics (22) by overcoming resistance mechanisms and modulating the tumor microenvironment (23). Our recent work demonstrated that in a lysosomal pH–dependent manner, (H)CQ sensitizes specific ovarian cancer cell lines to ITRA with Bliss-defined cytotoxic synergy observed (24). Given the dependencies on cholesterol homeostasis in both prostate cancer and ovarian cancer (25, 26), we hypothesized that SI also synergizes with HCQ in prostate cancer and this mechanism may be particularly potent in androgen-sensitive prostate cancer in which cholesterol is destined for androgen synthesis (27). We also undertook translational studies to explore lipidomic biomarkers of activity given recent findings suggesting the importance of lysosome function to maintain cholesterol homeostasis supporting intratumoral androgen synthesis and energy production via β-oxidation (28–31), which is linked to prostate cancer progression and therapeutic resistance (32). Multiple studies have highlighted the lipid-modulatory effects of ITRA and HCQ. ITRA inhibits cholesterol biosynthesis (14) and disrupts cholesterol trafficking by binding to and inhibiting Niemann-Pick C1 (NPC1), a key lysosomal cholesterol transporter (33–35). In preclinical and clinical models, ITRA has been shown to reduce serum levels of total cholesterol (TC), triglycerides (TG), and low-density lipoprotein cholesterol (LDL-C), while increasing high-density lipoprotein cholesterol (36, 37). HCQ-induced lysosomal deacidification can impair cholesterol ester hydrolysis and trafficking, reducing LDL-C levels in some studies (38, 39).

To further investigate these possibilities, we conducted a series of preclinical studies evaluating the combination in prostate cancer cell line models. These studies, detailed herein, revealed significant synergistic cytotoxicity and provided the foundational rationale for the HITMAN-PC clinical trial, the phase I component of which is presented below.

## Patients and Methods

### Cell culture

LNCap (RRID: CVCL_0395) and paired enzalutamide-sensitive and enzalutamide-resistant LNCaP derivative cell lines V16D (castration-resistant prostate cancer) and MR-40C (enzalutamide-resistant prostate cancer) were kindly provided by Dr. Amina Zoubeidi (Vancouver Prostate Centre, University of British Columbia, BC, Canada) and derived as described previously (40). 22rv1 (RRID: CVCL_1045), DU145 (RRID: CVCL_0105), and PC3 (RRID: CVCL_0035) were obtained from ATCC. All cell lines were cultured in RPMI-1640 medium (Gibco) containing 10% FBS (Gibco) at 37°C in a humidified incubator with 5% CO_2_ for a maximum of 10 passages. Regular cell line authentication was done at The Centre for Applied Genomics (http://www.tcag.ca) using the GenePrint 10 System (Promega Corporation) according to the manufacturer’s instructions. All cell lines were routinely tested to confirm the absence of *Mycoplasma* using the MycoAlert Detection Kit (Lonza).

### Alamar Blue assay

Cell viability was assessed using the Alamar Blue assay, following the manufacturer’s protocol. Briefly, cells were seeded in 96-well plates at a density of 4 × 10^3^ cells/100 μL per well for LNCaP, V16D, MR-40C, and 22Rv1 and 2 × 10^3^ cells/100 μL per well for PC3 and DU145. After allowing cells to adhere overnight, they were treated with serial dilutions of ITRA (Sigma) at final concentrations ranging from 0 to 40 μmol/L, with or without CQ or HCQ (Sigma) at 5 to 10 μmol/L. After 5 days of treatment, Alamar Blue solution was added to each well, followed by 4-hour incubation. Absorbance was measured using a microplate reader (FLUOstar Omega, BMG Labtech) at a test wavelength of 550 nm. All experiments were performed independently in triplicate. Representative images were captured using the Incucyte SX5 live-cell imaging system (Sartorius).

### Synergy score calculation

Drug combination data were analyzed using SynergyFinder Plus (http://www.synergyfinderplus.org/; RRID: SCR_019318) as previously reported (41). The Bliss independence model was applied to calculate synergy scores across the dose–response matrix, and synergy maps were visualized using the platform’s built-in three-dimensional plotting tools.

### Statistical analysis

All dose–response graphs were generated using GraphPad Prism v10.4.2 (RRID: SCR_002798). Statistical significance of differences in synergy score groups was assessed using the unpaired *t* test or one-way ANOVA.

### Study design of HITMAN-PC (ClinicalTrials.govNCT03513211)

A phase I/II open-label, single-arm study employing a rolling-six phase I design was used to assess the combination of SI (Mayne Pharma) and HCQ (Generic Health Pty Ltd; bought commercially) in men with BCR prostate cancer. This report focuses on the completed phase I dose-escalation component. The study was conducted under the approval of the Institutional Review Board and in strict accordance with established ethical guidelines, including the Declaration of Helsinki. All participants provided written informed consent prior to study enrollment and any study-related procedures. The planned phase II component was designed as a Simon two-stage cohort expansion to further evaluate efficacy at the recommended phase II dose (RP2D). However, because of a significant change in the therapeutic landscape for BCR prostate cancer following the results of the EMBARK trial, and preliminary futility signals observed during the phase I component, the decision was made not to proceed with the phase II expansion. Therefore, accrual was completed after the phase I dose escalation.

Men received a fixed dosage of SI 150 mg twice daily orally with food. HCQ was administered orally twice daily, as per a dose-escalation schedule: Dose level (DL) 1: 200 mg twice daily; DL2: 400 mg twice daily; and DL3: 600 mg twice daily, continuously in a 28-day cycle. The fixed dose of SI was based on its enhanced bioavailability achieving equivalent exposures to higher doses of generic ITRA previously studied (18, 42). Given relative safety of both compounds and no known pharmacokinetic (PK) interactions or significantly overlapping toxicities at starting doses (43–45), a rolling-six design was incorporated.

Dose-limiting toxicities (DLT) included hematologic toxicities such as absolute neutrophil count (ANC) <0.5 × 10^9^/L, febrile neutropenia (ANC <1 × 10^9^/L with fever >38.5°C), or platelets <50 × 10^9^/L; specific nonhematologic toxicities like grade ≥3 diarrhea (despite loperamide) or grade ≥3 rash (or grade 2 if medically concerning/unacceptable); any other grade ≥3 treatment-related adverse event (TRAE; excluding manageable hypertension); or missing ≥21 days of treatment due to drug toxicity. Additionally, any ocular toxicity ≥ grade 2, including but not limited to retinopathy, constituted a DLT. DLTs were assessed during the initial 28-day treatment cycle at each new DL (protocol in Supplementary Materials and Methods). The planned sample size for the phase I component ranged from six to 18 patients.

Efficacy assessments primarily involved serial monitoring of PSA levels, which were measured at baseline and then prior to each 28-day treatment cycle. Radiographic staging, including CT of the chest, abdomen, and pelvis and a whole-body bone scan, was performed at baseline to confirm eligibility with regard to metastatic disease and repeated as clinically indicated to assess for disease progression or at the end of treatment. Toxicity was assessed using the NCI Common Terminology Criteria for Adverse Events version 5.0. To investigate the metabolic impact of the study treatment, plasma samples were collected for comprehensive lipidomic profiling.

The primary objective of the phase I component was the establishment of the MTD and determination of the RP2D. A key secondary objective was to characterize the safety and tolerability profile of the SI and HCQ combination in this patient population.

Exploratory efficacy objectives included the assessment of PSA response rate, defined as a ≥50% decrease in PSA from baseline. The median PSA-PFS was a key exploratory endpoint. For the primary reporting of PSA-PFS in this cohort, PSA progression was defined according to modified Prostate Cancer Clinical Trials Working Group 3 (PCWG3) criteria: the first PSA increase that is ≥25% and ≥2 ng/mL above the nadir, confirmed by a second value ≥3 weeks later. Given the absence of universally robust guidelines for outcome measures specifically in BCR, and acknowledging that PCWG3 guidelines predominantly apply to castration-resistant disease, the protocol also prespecified sensitivity analyses. These analyses involved defining PSA progression through alternative criteria: (i) first PSA increase ≥25% above nadir, confirmed by a second increase ≥3 weeks later; (ii) first PSA increase ≥50% above nadir, confirmed by a second increase ≥3 weeks later; and (iii) first PSA increase ≥25% above and ≥1 ng/mL above nadir, confirmed by a second increase ≥3 weeks later. Further exploratory efficacy objectives included the proportion of patients free of PSA progression at the 6-month landmark, changes in PSADT from baseline at 4 and 12 weeks of treatment, time to ADT commencement, and MFS and change in lipidomic profile at baseline and after cycle 2.

### Patients

Enrolled patients were adult men (≥18 years) with an Eastern Cooperative Oncology Group performance status of 0 to 1 and histologically confirmed adenocarcinoma of the prostate. Participants had BCR, characterized by PSA level ≥1 ng/mL that had increased on at least two sequential occasions measured at least 1 week apart, following prior definitive local therapy (RP or RT) and in the absence of widespread metastatic disease (by conventional imaging).

Further key eligibility criteria included serum testosterone ≥5 nmol/L, adequate organ function [including hematologic, hepatic, and renal parameters; e.g., ANC ≥1.5 × 10^9^/L, alanine aminotransferase (ALT)/aspartate aminotransferase (AST) <2 × upper limit of normal, and creatinine clearance >50 mL/minute], and a QTc ≤470 millisecond.

Major exclusion criteria comprised evidence of metastatic disease beyond protocol-defined low-volume limits, a PSADT ≤3 months, known G6PD deficiency, preexisting retinopathy predisposing to HCQ toxicity, significant cardiac comorbidities, or use of strong CYP3A4-interacting medications or contraindicated statins. Full eligibility criteria are detailed in the study protocol.

### Clinical trial exploratory biomarker analyses

Peripheral blood samples were prospectively collected at prespecified timepoints for PK analysis and comprehensive lipidomic profiling. Archival tumor tissue (from prior RP or diagnostic biopsies) was also collected for planned genomic analyses.

### Plasma PK analyses

Plasma concentrations of SI and HCQ were determined from samples collected at trough prior to dosing on day 1 of each cycle.

### Plasma lipidomic profiling

Plasma samples for lipidomic analysis were collected at baseline (cycle 1 day 1) and after approximately two cycles of treatment (cycle 3 day 1). Following extraction, untargeted lipidomic profiling was performed as described previously (46), quantifying up to 769 lipids (pmol/mL). Raw lipid abundance data underwent normalization and were log_2_ transformed for statistical analyses as described previously (47). Differential lipid analysis between baseline and posttreatment samples was conducted using limma (48) for paired samples, and lipid fold changes were calculated.

### Statistical analysis

Patient clinical features and response details are described using summary statistics, such as medians, ranges, frequencies, and proportions. PFS analysis was conducted using the Kaplan–Meier method for all patients. Median and confidence interval (CI) were reported to assess PFS. Treatment-related toxicity was evaluated using frequencies and proportions of adverse events based on severities and attributions.

## Results

### Preclinical evaluation of ITRA and CQ/HCQ in prostate cancer cell lines

To investigate the preclinical rationale for combining ITRA with a lysosomotropic agent, we evaluated the cytotoxic effects of ITRA alone and in combination with (H)CQ across a panel of human prostate cancer cell lines. For this purpose, we used a panel of cell lines sharing the same genetic background, all derived from LNCaP, representing different stages of prostate cancer progression: LNCaP (hormone sensitive), V16D (castration resistant), and MR-40C (enzalutamide resistant). Additionally, we included castration- and enzalutamide-resistant cell lines with distinct genetic backgrounds: PC3 and DU145 [androgen receptor (AR) null] and 22Rv1 (expressing the AR splice variant AR-V7).

**Figure 1.**
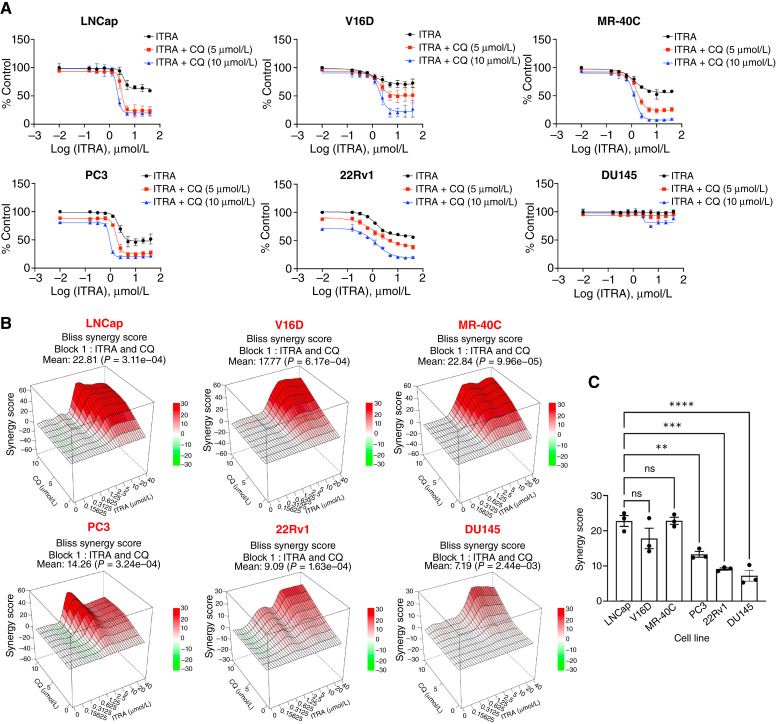
Synergistic cytotoxicity of ITRA and CQ in prostate cancer cell lines. **A,** Dose–response curves showing the activity of ITRA alone (black) or in combination with 5 μmol/L (red) and 10 μmol/L (blue) CQ in a panel of six prostate cancer cell lines after 5 days of treatment. **B,** Representative three-dimensional synergy maps showing the combined effect of ITRA (0–40 μmol/L) and CQ (5–10 μmol/L) in a panel of six prostate cancer cell lines. **C,** Histogram plot showing the synergy scores calculated using a Bliss independence model of combination of ITRA and CQ (*n* = 3 biological replicates, **, *P* < 0.01; ***, *P* < 0.001; ****, *P* < 0.0001; ns, not significant).

ITRA monotherapy exhibited varying degrees of dose-dependent growth inhibition in the cell lines tested, including LNCap, V16D, MR-40C, 22Rv1, DU145, and PC3 (Fig. 1A; Supplementary Fig. S1). PC3 cells exhibited more pronounced sensitivity, whereas LNCaP, 22Rv1, and MR-40C showed moderate responses. In contrast, V16D and DU145 seemed more resistant within the tested concentration range.

Co-treatment with fixed concentrations of CQ (5 and 10 μmol/L) generally enhanced the cytotoxic activity of ITRA in several of the cell lines. This potentiation was particularly evident in LNCaP, V16D, and MR-40C cells, in which the addition of CQ led to a greater reduction in cell viability compared with ITRA alone, especially at higher drug concentrations, and was reflected in the synergy score calculations (Fig. 1B and C). LNCaP and its enzalutamide-sensitive and -resistant derivatives (V16D and MR-40C) exhibited comparable synergy scores, with no statistically significant differences among them. In contrast, PC3, 22Rv1, and DU145 showed significantly lower synergy scores compared with LNCaP, indicating reduced sensitivity to the ITRA/CQ combination, with DU145 being the least responsive.

Microscopic examination of cells treated with ITRA alone or in combination with CQ further supported the viability data, revealing more pronounced effects on cell density and morphology with the combination treatment compared with either monotherapy or the DMSO vehicle control. These effects were most evident in cell lines exhibiting the highest synergy scores, such as LNCap and MR-40C (Supplementary Fig. S1).

To confirm that HCQ would elicit similar preclinical effects, its combination with ITRA was also tested. A comparable enhancement of ITRA’s cytotoxic effect was observed when combined with HCQ (5–10 μmol/L) in LNCap, V16D, and MR-40C cell lines, suggesting similar sensitizing activity between CQ and HCQ in this preclinical setting (Supplementary Figs. S2 and S3).

### Clinical trial results: phase I dose escalation of SI and HCQ (HITMAN-PC)

#### Baseline characteristics

Based on the promising preclinical observations, the HITMAN-PC phase I dose-escalation study was conducted. Between 2018 and 2019, a total of 11 men with BCR prostate cancer were enrolled and treated. All patients received at least one dose of study medication and were evaluable for safety and DLT assessment.

Baseline patient and disease characteristics for the enrolled cohort are summarized in Table 1, and a comparison of these demographics with the broader disease population is provided in Supplementary Table S1.

**Table 1.** Patient characteristics.

Characteristic	*N* = 11^a^
Age	73 (69–77)
Baseline PSA (ng/mL)	4.4 (1.6–22.4)
Baseline PSADT (months)	5.31 (3.34–15.28)
Gleason score (ISUP^b^ grade group)	​
3 + 4 (grade group 2)	4 (36%)
4 + 3 (grade group 3)	2 (18%)
8 (grade group 4)	2 (18%)
9 (grade group 5)	2 (18%)
Unknown	1
Previous local treatment	​
RP + salvage RT	7 (64%)
RP alone	2 (18%)
RT alone	2 (18%)
ECOG performance status	​
0	10 (91%)
1	1 (9.1%)

Abbreviation: ECOG, Eastern Cooperative Oncology Group.

a Median (min–max); n (%).

b ISUP, International Society of Urological Pathology.

#### Dosage, safety, and clinical activity

Patients were treated across three DLs of HCQ (DL1: 200 mg twice daily; DL2: 400 mg twice daily; and DL3: 600 mg twice daily) in combination with SI 150 mg twice daily. Two DLTs occurred at DL3: one instance of grade 3 diarrhea and one of grade 3 ALT elevation. No DLTs were observed at DL1 or DL2. The MTD was thus HCQ 600 mg twice daily with SI 150 mg twice daily.

The safety profile is detailed in Table 2.

**Table 2.** Summary of TRAEs (N = 11).

Toxicity, no. (%)	Grade 1–2	Grade 3
Any toxicity	11 (100)	6 (55)
Hypertension	8 (73)	2 (18)
Raised QTc	6 (55)	0 (0)
Fatigue	4 (36)	0 (0)
Nausea	4 (36)	0 (0)
Diarrhea	3 (27)	1 (9)
Visual disturbance	4 (36)	0 (0)
ALT or AST increase	3 (27)	1 (9)

Serious adverse event 2: NSTEMI and volvulus, both unrelated to study treatments.

All patients experienced at least one TRAE. The most frequently reported TRAEs were hypertension (91%), QTc prolongation (55%), diarrhea (36%), and nausea (36%). Grade 3 TRAEs included hypertension (18%), diarrhea (9%), and ALT/AST elevation (9%). No grade 4 TRAEs or treatment-related deaths occurred. Two serious adverse events (NSTEMI and volvulus) were reported but considered unrelated to the study treatment.

**Figure 2.**
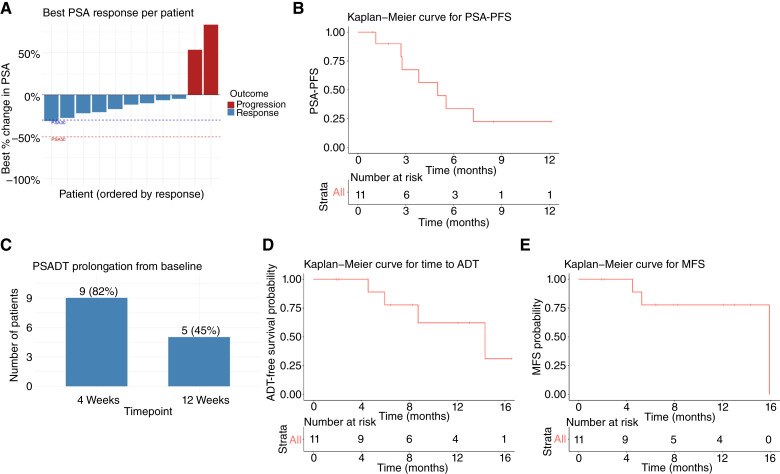
Clinical activity and time-to-event endpoints. **A,** Waterfall plot of the maximum percentage change in PSA from baseline. **B,** Kaplan–Meier analysis of PSA-PFS. **C,** Bar chart showing the proportion of patients with prolongation of PSADT at 4 and 12 weeks. **D** and **E,** Kaplan–Meier analyses of (**D**) time to ADT and (**E**) MFS.

No confirmed PSA responses (≥50% decline from baseline) were observed. However, nine of 11 (82%) patients experienced some decline in PSA levels from baseline, with a maximum observed decline of 31% (Fig. 2A). The median PSA-PFS was 5.5 months (95% CI, 2–9 months; Fig. 2B). At the 6-month landmark, six of 11 (54.5%) patients remained free of PSA progression. PSADT was prolonged from baseline at 4 and 12 weeks in nine (82%) and five (45%) patients, respectively (Fig. 2C). The median time to ADT commencement was 14.3 months (95% CI, 4.9–23.8 months; Fig. 2D). The median MFS was 15.9 months (95% CI, unevaluable; Fig. 2E).

#### PK

Plasma concentrations of HCQ and SI were measured at multiple timepoints throughout the study from all 11 enrolled patients. Steady-state HCQ concentrations were estimated by averaging values from samples collected after day 88 of treatment, based on an assumed half-life of approximately 22 days. For SI, estimated steady-state concentrations were derived by averaging measurements from cycle 2 to cycle 4, with exclusion of identified outliers.

For HCQ, the mean (±SD) estimated steady-state concentration across the cohort was 191 ± 122.8 ng/mL, with a median of 198 ng/mL (range, 0–411.2 ng/mL). For SI, the mean (±SD) estimated steady-state concentration was 1,439.7 ± 576.1 ng/mL, with a median of 1,669 ng/mL (range, 581–2,203 ng/mL). Considerable interpatient variability was observed in the steady-state concentrations for both drugs as indicated by the wide ranges and large SDs relative to the means.

#### Exploratory PK/pharmacodynamic analyses

To explore potential relationships between systemic drug exposure and clinical activity, patients were dichotomized based on their estimated individual steady-state concentrations of HCQ and SI.

**Figure 3.**
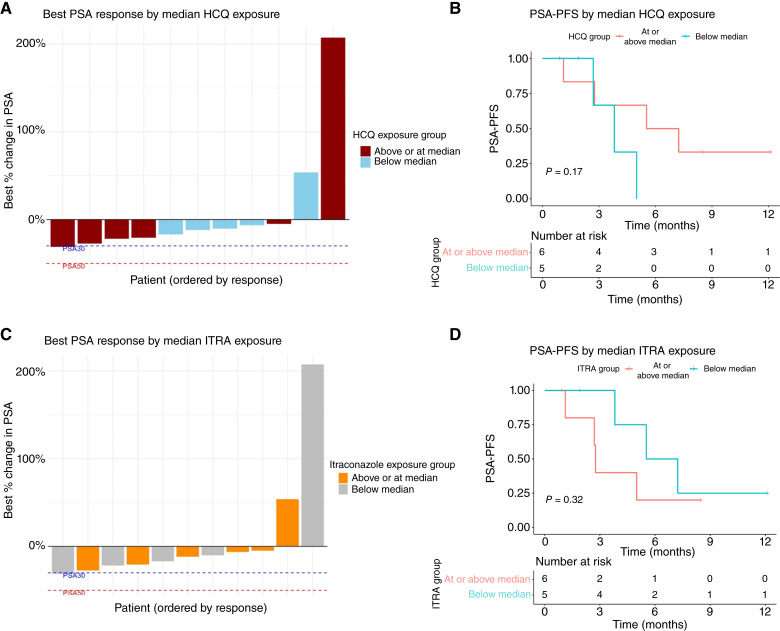
Correlation of steady-state drug exposure with clinical activity. **A,** Waterfall plot of best PSA response stratified by median steady-state HCQ exposure. **B,** Kaplan–Meier estimates of PSA-PFS stratified by median HCQ exposure. **C,** Waterfall plot of best PSA response stratified by median steady-state SI (ITRA) exposure. **D,** Kaplan–Meier estimates of PSA-PFS stratified by median SI exposure.

Patients were stratified by median steady-state HCQ levels (above vs. at/below the cohort median of 198 ng/mL). Waterfall plots illustrating best percentage change in PSA from baseline suggested a trend toward greater PSA declines in some patients with higher HCQ exposure although this was not uniformly observed (Fig. 3A).

Exploration of PSA-PFS showed a numerical trend toward longer median PSA-PFS in patients with at-or-above-median steady-state HCQ levels [6.38 months; 95% CI, 2.76–not applicable (NA)] compared with those with below-median levels (3.81 months; 95% CI, 2.70–NA). However, this trend did not reach statistical significance (*P* = 0.17, log-rank test; Fig. 3B).

Similarly, patients were stratified based on their estimated steady-state SI concentrations (at/below vs. above the cohort median of 1,669 ng/mL). Visual inspection of the corresponding waterfall plot also showed no clear correlation between higher SI exposure and the magnitude of PSA response; patients with both high and low SI exposure were observed across the spectrum of PSA responses (Fig. 3C).

A Kaplan–Meier analysis of PSA-PFS revealed a median PSA-PFS of 2.76 months (95% CI, 2.70–NA) in patients with at-or-above-median SI levels, compared with a numerically longer median PSA-PFS of 6.38 months (95% CI, 3.81–NA) in those with below-median levels. This observed difference did not reach statistical significance (*P* = 0.32, log-rank test; Fig. 3D).

#### Exploratory lipidomic analyses

To investigate the impact of treatment with SI and HCQ on systemic metabolism and to identify potential lipid biomarkers associated with clinical outcomes, comprehensive lipidomic profiling was performed on plasma samples. Paired samples from 11 patients were initially available; however, two patients were excluded from all lipidomic analyses because of early treatment discontinuation resulting from toxicity, with their treated plasma reflecting only 1 month of therapy. Thus, analyses were conducted on paired baseline and posttreatment plasma samples from nine evaluable patients. Based on the known mechanisms of the study drugs, disruption of lysosomal cholesterol transport by SI (13) and inhibition of lysosomal function by HCQ (20), we hypothesized that treatment would lead to specific alterations in sterol metabolism and complex lipids processed through the lysosome, such as sphingolipids.

**Figure 4.**
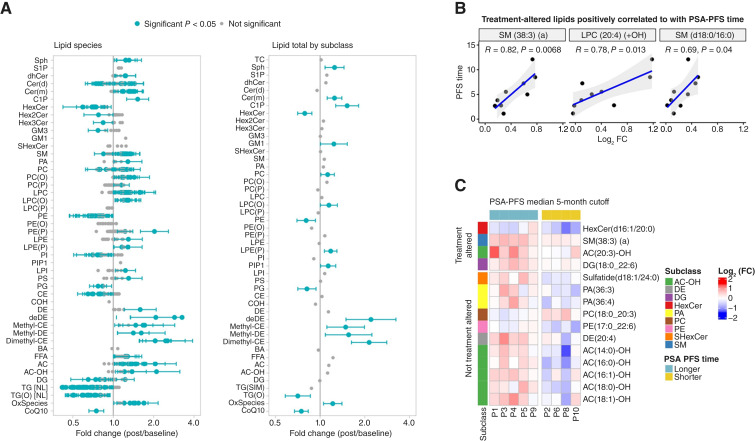
Effect of HCQ + SI treatment on the plasma lipidome. **A,** Average fold change (posttreatment/baseline) of individual lipid species. **B,** Scatter plots showing a significant positive correlation between the fold change of key treatment-altered lipid species and PSA-PFS time. **C,** Heatmap of the fold change (posttreatment/baseline) for lipids that are significantly different between patients with longer (>5 months) vs. shorter (≤5 months) PSA-PFS time. AC, acylcarnitines; AC-OH, hydroxylated acylcarnitines; BA, bile acids; CE, cholesteryl esters; Cer, ceramides; CIP, ceramide phosphates; CoQ10, coenzyme Q10; DE, dehydrocholesteryl esters; deDE, dehydrodesmosteryl esters; DG, diacylglycerols; FFA, free fatty acids; HexCer, hexosylceramides; LPC(O), lysophosphatidylcholines with an O-alkyl chain; LPC, lysophosphatidylcholines; LPE, lysophosphatidylethanolamines; LPI, lysophosphatidylinositols; OxSpecies, oxidized lipid species; PA, phosphatidic acids; PC, phosphatidylcholines; PE, phosphatidylethanolamines; PG, phosphatidylglycerols; PI, phosphatidylinositols; PS, phosphatidylserines; SHexCer, sulfatides (sulfated hexosylceramides); SM/Sph, sphingomyelins; TG(O), ether-linked triacylglycerols; TG, triacylglycerols.

Notably, sterols exhibited some of the largest fold changes. All four measured dimethyl-cholesteryl ester (dimethyl-CE) species were significantly increased after treatment and were among the top 20 most significantly treatment-altered individual lipids (Fig. 4A). Although dehydrodesmosteryl ester (deDE) species also showed large fold increases, their variability was high. Free cholesterol and TC ester (CE) levels were not significantly altered overall although several individual CE species were decreased, reflecting a complex disruption of cholesterol homeostasis (Fig. 4A). The analysis also supported the second part of our hypothesis, revealing significant changes in complex lipids associated with lysosomal processing; lipid species with increased levels were significantly enriched for sphingomyelins (Sph) and ceramides with an m-hydroxy fatty acid, in addition to acylcarnitines (AC) and several cholesterol-related lipids such as dimethyl-CE and deDE, whereas those with decreased levels were enriched for hexosylceramides, as well as the most significantly altered sub-classes, TG and ether-linked triacylglycerols [TG(O); Fisher exact test *P* < 0.05; Supplementary Table S2].

An unbiased, global analysis identified that a total of 239 of 769 distinct lipid species belonging to 46 lipid subclasses were significantly altered by treatment (*P* < 0.05) across the nine evaluable patients. Of these, 121 showed increased levels and 118 showed decreased levels after treatment compared with baseline. Similarly, at the subclass level, the total levels of 13 lipid subclasses were significantly increased, whereas five were decreased (Fig. 4A). Beyond the sterol and sphingolipid classes, other major alterations included a significant decrease in TG and TG(O) and phosphatidylethanolamines and an increase in oxidized lipid species and lysophosphatidylcholines with an O-alkyl chain (Fig. 4A; Supplementary Table S2).

An exploratory analysis was conducted to examine whether different HCQ DLs (low: 200 mg twice daily; mid: 400 mg twice daily; and high: 600 mg twice daily) differentially affected the magnitude of change in treatment-altered lipids. Of the 239 lipids altered by treatment, 24 showed significantly different fold changes between mid- versus low-dose groups, 35 between high- versus low-dose groups, and nine between high- versus mid-dose groups. Interestingly, patients in the lowest HCQ dose group (200 mg twice daily) exhibited a larger magnitude of change for certain treatment-altered lipids, such as increases in AC and dimethyl-CE and decreases in TG(O) and TG, compared with patients receiving mid or high HCQ doses (Supplementary Fig. S4). However, the small number of patients, particularly in the high-dose group (*n* = 2 for lipidomic analysis), limited the ability to confidently identify robust dose-dependent effects.

Pearson correlation analysis identified 34 plasma lipids for which fold change significantly correlated with PSA-PFS time (*P* < 0.05). Among the 11 of these that were also treatment-altered lipids, a decrease in the fold change of several TG species was associated with longer PSA-PFS (Supplementary Fig. S5A). This is consistent with treatment generally decreasing TG species. Conversely, an increase in the fold change of the treatment-altered Sph SM(38:3)(a) was also significantly correlated with longer PSA-PFS (R = 0.82; *P* = 0.0068; Fig. 4B). The total levels of all SM lipids also showed a positive correlation with PSA-PFS (R = 0.74; *P* = 0.024; Supplementary Fig. S5B).

Further analysis comparing lipid fold changes between patient groups with longer versus shorter PSA-PFS (using a median PSA-PFS cutoff of 5 months) showed that patients with longer PSA-PFS had significantly increased levels of 7 AC-OH (hydroxylated AC) species after treatment (of which one was treatment altered and six were non–treatment altered) compared with those with shorter PSA-PFS [e.g., AC(20:3)-OH; Fig. 4C]. Similar, although less extensive, differences in AC-OH species were observed with other PSA-PFS cutoffs (Supplementary Fig. S6A and S6B).

## Discussion

The HITMAN-PC study was designed to explore the therapeutic potential of combining SI with HCQ in men with BCR prostate cancer, a disease state with limited nonhormonal treatment options. The combination demonstrated a manageable safety profile, establishing an RP2D of SI 150 mg twice daily with HCQ 600 mg twice daily. Although definitive antitumor responses, e.g., PSA50 responses, were not achieved, the treatment demonstrated biological activity as evidenced by PSA stabilization or decline in nine of 11 (82%) patients, with a maximum observed decline from baseline of 31%. However, the modest median PSA-PFS of 5.5 months indicates that this approach, as tested, is unlikely to offer durable clinical benefit. These findings highlight the ongoing difficulty of translating promising preclinical synergy into meaningful patient outcomes, a recurring challenge in metabolism-targeted therapies.

Despite the lack of clear antitumor efficacy, the study provides important mechanistic insights. Plasma lipidomic profiling revealed significant and pathway-specific alterations induced by the HCQ and SI combination, supporting a proof of biology for this therapeutic approach. The observed decrease in multiple TG species and the increase in Sph SM(38:3)(a), and the correlation of these changes with PSA-PFS, point to a direct link between treatment-induced metabolic rewiring and clinical outcomes. For instance, the alterations in Sphs are consistent with the hypothesized disruption of lysosomal function by HCQ, whereas the changes in TGs may reflect broader effects on cellular energy metabolism. These observations indicate that the systemic metabolic consequences of targeting cholesterol homeostasis and autophagy can be captured in real time, providing a deeper understanding of the drug combination’s biological impact in patients.

The combination of SI and HCQ demonstrated a manageable but notable toxicity profile. The most frequent grade 3 adverse events, hypertension, diarrhea, and ALT elevation, are known potential side effects of the individual agents, particularly the mineralocorticoid excess syndrome associated with ITRA (24, 49, 50). Although these toxicities were manageable in this phase I setting and led to the establishment of the RP2D, they represent a significant challenge for broader clinical development, especially for men who are asymptomatic in the biochemically recurrent setting. Going forward, managing these toxicities would likely require proactive strategies. For example, the ITRA-induced hypertension and hypokalemia might be mitigated with mineralocorticoid receptor antagonists like spironolactone or eplerenone (51, 52). Prophylactic antidiarrheal regimens could also be considered. However, these toxicities would need to be considered in the context of now established treatment paradigms that are better tolerated such as in the EMBARK trial (11).

Several limitations must be acknowledged. The small sample size and single-arm design restrict generalizability, whereas the absence of a control group precludes firm conclusions about whether the lipidomic alterations were treatment specific or reflective of disease biology. Furthermore, although exploratory lipidomic analyses revealed associations with PSA-PFS that reached nominal significance in this dataset, these findings must be interpreted with caution and validated in larger, prospectively designed studies.

Ultimately, the evolving therapeutic landscape in BCR prostate cancer, particularly following the EMBARK trial, together with the modest efficacy signals observed in HITMAN-PC, precluded progression to the planned phase II expansion. Nonetheless, this study provides a valuable foundation by characterizing the safety, PK, and metabolic effects of SI and HCQ in this setting. Future research should build upon these insights by validating lipidomic signatures as predictive or pharmacodynamic biomarkers and by integrating metabolism-targeting strategies into rationally designed clinical trials.

### Conclusion

In this phase I evaluation, the combination of SI and HCQ demonstrated a manageable safety profile but lacked evidence of meaningful clinical efficacy in men with BCR prostate cancer. Nonetheless, the integration of PK and lipidomic analyses provided valuable proof-of-biology insights, revealing treatment-induced alterations in cholesterol and autophagy-related pathways and identifying candidate plasma lipids associated with outcomes. Although this regimen will not advance further, the study highlights the importance of embedding translational endpoints into early-phase trials, ensuring that even negative results inform biomarker discovery and deepen our understanding of prostate cancer biology.

## Supplementary Material

Supplementary Figure 1 Representative images of six prostate cancer cell lines treated with Itraconazole alone or in combination with chloroquine (CQ) for 5 days.

Supplementary Figure 2 Dose-response curves, 3D synergy maps, and histograms comparing the combined cytotoxic effect of Itraconazole with CQ or HCQ in LNCaP, V16D, and MR-40C cells.

Supplementary Figure 3 Representative images of LNCaP, V16D, and MR-40C cells treated with Itraconazole alone or in combination with CQ or HCQ for 5 days

Supplementary Figure 4 Heatmap showing the fold change of treatment-altered lipids that exhibited significantly different magnitudes of change across the three HCQ dose groups.

Supplementary Figure 5 Scatter plots demonstrating significant correlations between the fold change of specific treatment-altered lipids (triacylglycerols, oxidized species, and sphingomyelins) and PSA-PFS time.

Supplementary Figure 6 Heatmaps comparing plasma lipid fold changes between patient groups defined by alternative PSA-PFS time cutoffs.

Supplementary Table 1 Detailed enrichment analysis showing the differential lipid species and total lipid species altered post-treatment by sub-class.

Supplementary Table 2 Comparison of the study participants' demographic characteristics with the general population of men with biochemically recurrent prostate cancer.

## Data Availability

Because of patient privacy concerns and institutional ethics constraints with regard to the clinical trial, the raw data cannot be deposited on the Vivli platform. Deidentified data may be made available upon reasonable request to the corresponding author.

## References

[1] Sung H , Ferlay J, Siegel RL, Laversanne M, Soerjomataram I, Jemal A, . Global cancer statistics 2020: GLOBOCAN estimates of incidence and mortality worldwide for 36 cancers in 185 countries. CA Cancer J Clin 2021;71:209–49. 33538338 10.3322/caac.21660

[2] Surveillance, Epidemiology, and End Results Program . Cancer of the prostate - cancer stat facts. Bethesda (MD): National Cancer Institute; n.d. [cited 2024 Jan 30]. Available from: https://seer.cancer.gov/statfacts/html/prost.html.

[3] Sciarra A , Santarelli V, Salciccia S, Moriconi M, Basile G, Santodirocco L, . How the management of biochemical recurrence in prostate cancer will be modified by the concept of anticipation and incrementation of therapy. Cancers 2024;16:764. 38398155 10.3390/cancers16040764 PMC10886975

[4] Stensland KD , Caram MV, Burns JA, Sparks JB, Shin C, Zaslavsky A, . Recurrence, metastasis, and survival after radical prostatectomy in the era of advanced treatments. J Clin Oncol 2022;40(16_suppl):5090.

[5] Marshall CH , Chen Y, Kuo C, Cullen J, Jiang J, Rosner I, . Timing of androgen deprivation treatment for men with biochemical recurrent prostate cancer in the context of novel therapies. J Urol 2021;206:623–9. 34003011 10.1097/JU.0000000000001797 PMC9774680

[6] Karim MU , Tisseverasinghe S, Cartes R, Martinez C, Bahoric B, Niazi T. Early versus delayed androgen deprivation therapy for biochemical recurrence after local curative treatment in non-metastatic hormone-sensitive prostate cancer: a systematic review of the literature. Cancers (Basel) 2025;17:215. 39857997 10.3390/cancers17020215 PMC11764282

[7] Higano CS . Side effects of androgen deprivation therapy: monitoring and minimizing toxicity. Urology 2003;61:32–8. 12667885 10.1016/s0090-4295(02)02397-x

[8] Loblaw A , Bassett J, D’Este C, Pond GR, Cheung P, Millar JL, . Timing of androgen deprivation therapy for prostate cancer patients after radiation: planned combined analysis of two randomized phase 3 trials. J Clin Oncol 2018;36:5018.

[9] Deek MP , Van der Eecken K, Sutera P, Deek RA, Fonteyne V, Mendes AA, . Long-term outcomes and genetic predictors of response to metastasis-directed therapy versus observation in oligometastatic prostate cancer: analysis of STOMP and ORIOLE trials. J Clin Oncol 2022;40:3377–82. 36001857 10.1200/JCO.22.00644 PMC10166371

[10] Phillips R , Shi WY, Deek M, Radwan N, Lim SJ, Antonarakis ES, . Outcomes of observation vs stereotactic ablative radiation for oligometastatic prostate cancer: the ORIOLE phase 2 randomized clinical trial. JAMA Oncol 2020;6:650–9. 32215577 10.1001/jamaoncol.2020.0147 PMC7225913

[11] Freedland SJ , de Almeida Luz M, de Giorgi U, Gleave M, Gotto GT, Pieczonka CM, . Improved outcomes with enzalutamide in biochemically recurrent prostate cancer. N Engl J Med 2023;389:1453–65. 37851874 10.1056/NEJMoa2303974

[12] Xia Y , Sun M, Huang H, Jin W-L. Drug repurposing for cancer therapy. Signal Transduct Target Ther 2024;9:92. 38637540 10.1038/s41392-024-01808-1 PMC11026526

[13] Pantziarka P , Sukhatme V, Bouche G, Meheus L, Sukhatme VP. Repurposing Drugs in Oncology (ReDO)–itraconazole as an anti-cancer agent. Ecancermedicalscience 2015;9:521. 25932045 10.3332/ecancer.2015.521 PMC4406527

[14] Li C-L , Fang Z-X, Wu Z, Hou Y-Y, Wu H-T, Liu J. Repurposed itraconazole for use in the treatment of malignancies as a promising therapeutic strategy. Biomed Pharmacother 2022;154:113616. 36055112 10.1016/j.biopha.2022.113616

[15] Abuhelwa AY , Foster DJR, Mudge S, Hayes D, Upton RN. Population pharmacokinetic modeling of itraconazole and hydroxyitraconazole for oral SUBA-itraconazole and sporanox capsule formulations in healthy subjects in fed and fasted states. Antimicrob Agents Chemother 2015;59:5681–96. 26149987 10.1128/AAC.00973-15 PMC4538523

[16] Rauseo AM , Mazi P, Lewis P, Burnett B, Mudge S, Spec A. Bioavailability of single-dose SUBA-itraconazole compared to conventional itraconazole under fasted and fed conditions. Antimicrob Agents Chemother 2021;65:e0013421. 34031053 10.1128/AAC.00134-21 PMC8284459

[17] Lee M , Hong H, Kim W, Zhang L, Friedlander TW, Fong L, . Itraconazole as a noncastrating treatment for biochemically recurrent prostate cancer: a phase 2 study. Clin Genitourinary Cancer 2019;17:e92–6. 10.1016/j.clgc.2018.09.013 30327180

[18] Antonarakis ES , Heath EI, Smith DC, Rathkopf D, Blackford AL, Danila DC, . Repurposing itraconazole as a treatment for advanced prostate cancer: a noncomparative randomized phase II trial in men with metastatic castration-resistant prostate cancer. Oncologist 2013;18:163–73. 23340005 10.1634/theoncologist.2012-314 PMC3579600

[19] Rebecca VW , Amaravadi RK. Emerging strategies to effectively target autophagy in cancer. Oncogene 2016;35:1–11. 25893285 10.1038/onc.2015.99 PMC4838040

[20] Al-Bari MAA . Chloroquine analogues in drug discovery: new directions of uses, mechanisms of actions and toxic manifestations from malaria to multifarious diseases. J Antimicrob Chemother 2015;70:1608–21. 25693996 10.1093/jac/dkv018 PMC7537707

[21] Lin J-Z , Wang W-W, Hu T-T, Zhu G-Y, Li L-N, Zhang C-Y, . FOXM1 contributes to docetaxel resistance in castration-resistant prostate cancer by inducing AMPK/mTOR-mediated autophagy. Cancer Lett 2020;469:481–9. 31738958 10.1016/j.canlet.2019.11.014

[22] Saleem A , Dvorzhinski D, Santanam U, Mathew R, Bray K, Stein M, . Effect of dual inhibition of apoptosis and autophagy in prostate cancer. Prostate 2012;72:1374–81. 22241682 10.1002/pros.22487 PMC3840901

[23] Ashrafizadeh M , Paskeh MDA, Mirzaei S, Gholami MH, Zarrabi A, Hashemi F, . Targeting autophagy in prostate cancer: preclinical and clinical evidence for therapeutic response. J Exp Clin Cancer Res 2022;41:105. 35317831 10.1186/s13046-022-02293-6 PMC8939209

[24] Marastoni S , Madariaga A, Pesic A, Nair SN, Li ZJ, Shalev Z, . Repurposing itraconazole and hydroxychloroquine to target lysosomal homeostasis in epithelial ovarian cancer. Cancer Res Commun 2022;2:293–306. 36875717 10.1158/2767-9764.CRC-22-0037 PMC9981200

[25] He J , Siu MKY, Ngan HYS, Chan KKL. Aberrant cholesterol metabolism in ovarian cancer: identification of novel therapeutic targets. Front Oncol 2021;11:738177. 34820325 10.3389/fonc.2021.738177 PMC8606538

[26] Škara L , Huđek Turković A, Pezelj I, Vrtarić A, Sinčić N, Krušlin B, . Prostate cancer—focus on cholesterol. Cancers (Basel) 2021;13:4696. 34572923 10.3390/cancers13184696 PMC8469848

[27] Miller WL . Androgen biosynthesis from cholesterol to DHEA. Mol Cell Endocrinol 2002;198:7–14. 12573809 10.1016/s0303-7207(02)00363-5

[28] Ahmad F , Cherukuri MK, Choyke PL. Metabolic reprogramming in prostate cancer. Br J Cancer 2021;125:1185–96. 34262149 10.1038/s41416-021-01435-5 PMC8548338

[29] Zaidi N , Lupien L, Kuemmerle NB, Kinlaw WB, Swinnen JV, Smans K. Lipogenesis and lipolysis: the pathways exploited by the cancer cells to acquire fatty acids. Prog Lipid Res 2013;52:585–9. 24001676 10.1016/j.plipres.2013.08.005 PMC4002264

[30] Suburu J , Chen YQ. Lipids and prostate cancer. Prostaglandins Other Lipid Mediat 2012;98:1–10. 22503963 10.1016/j.prostaglandins.2012.03.003 PMC3348998

[31] Clarke N , Brown M. The influence of lipid metabolism on prostate cancer development and progression: is it time for a closer look? Eur Urol 2007;52:3–4. 17467164 10.1016/j.eururo.2007.04.039

[32] Parupathi P , Devarakonda LS, Francois E, Amjed M, Kumar A. Reprogrammed lipid metabolism-associated therapeutic vulnerabilities in prostate cancer. Int J Mol Sci 2025;26:9132. 41009695 10.3390/ijms26189132 PMC12470783

[33] Long T , Qi X, Hassan A, Liang Q, De Brabander JK, Li X. Structural basis for itraconazole-mediated NPC1 inhibition. Nat Commun 2020;11:152. 31919352 10.1038/s41467-019-13917-5 PMC6952396

[34] Trinh MN , Lu F, Li X, Das A, Liang Q, De Brabander JK, . Triazoles inhibit cholesterol export from lysosomes by binding to NPC1. Proc Natl Acad Sci U S A 2017;114:89–94. 27994139 10.1073/pnas.1619571114 PMC5224357

[35] Head SA , Shi WQ, Yang EJ, Nacev BA, Hong SY, Pasunooti KK, . Simultaneous targeting of NPC1 and VDAC1 by itraconazole leads to synergistic inhibition of mTOR signaling and angiogenesis. ACS Chem Biol 2017;12:174–82. 28103683 10.1021/acschembio.6b00849 PMC5791891

[36] Alsuwaidan S , Alajlan A, Alkreathy H. The effects of terbinafine on the lipid profile in humans and rabbits. Med Sci 2023;27:e185ms2947.

[37] Mohammad P , Amir F, Dorna A, Jahromi H. The inhibitory effect of Itraconazole on atherosclerosis in hyperlipidemic rabbits. Biosciences Biotechnol Res Asia 2014;11:1037–45.

[38] Migkos MP , Markatseli TE, Iliou C, Voulgari PV, Drosos AA. Effect of hydroxychloroquine on the lipid profile of patients with Sjögren syndrome. J Rheumatol 2014;41:902–8. 24634203 10.3899/jrheum.131156

[39] Babary H , Liu X, Ayatollahi Y, Chen XP, Doo L, Uppaluru LK, . Favorable effects of hydroxychloroquine on serum low density lipid in patients with systemic lupus erythematosus: a systematic review and meta-analysis. Int J Rheum Dis 2018;21:84–92. 28884965 10.1111/1756-185X.13159

[40] Toren P , Kim S, Johnson F, Zoubeidi A. Combined AKT and MEK pathway blockade in pre-clinical models of enzalutamide-resistant prostate cancer. PLoS One 2016;11:e0152861. 27046225 10.1371/journal.pone.0152861 PMC4821639

[41] Zheng S , Wang W, Aldahdooh J, Malyutina A, Shadbahr T, Tanoli Z, . SynergyFinder plus: toward better interpretation and annotation of drug combination screening datasets. Genomics Proteomics Bioinformatics 2022;20:587–96. 35085776 10.1016/j.gpb.2022.01.004 PMC9801064

[42] Suzman DL , Antonarakis ES. High-dose itraconazole as a noncastrating therapy for a patient with biochemically recurrent prostate cancer. Clin Genitourin Cancer 2014;12:e51–3. 24332506 10.1016/j.clgc.2013.11.015 PMC3959234

[43] Haria M , Bryson HM, Goa KL. Itraconazole. A reappraisal of its pharmacological properties and therapeutic use in the management of superficial fungal infections. Drugs 1996;51:585–620. 8706596 10.2165/00003495-199651040-00006

[44] Tett S , McLachlan A, Day R, Cutler D. Insights from pharmacokinetic and pharmacodynamic studies of hydroxychloroquine. Agents Actions Suppl 1993;44:145–90. 8372723

[45] Lee JY , Vinayagamoorthy N, Han K, Kwok SK, Ju JH, Park KS, . Association of polymorphisms of cytochrome P450 2D6 with blood hydroxychloroquine levels in patients with systemic lupus erythematosus. Arthritis Rheumatol 2016;68:184–90. 26316040 10.1002/art.39402

[46] Huynh K , Barlow CK, Jayawardana KS, Weir JM, Mellett NA, Cinel M, . High-throughput plasma lipidomics: detailed mapping of the associations with cardiometabolic risk factors. Cell Chem Biol 2019;26:71–84.e4. 30415965 10.1016/j.chembiol.2018.10.008

[47] Lin H-M , Mahon KL, Weir JM, Mundra PA, Spielman C, Briscoe K, . A distinct plasma lipid signature associated with poor prognosis in castration-resistant prostate cancer. Int J Cancer 2017;141:2112–20. 28741687 10.1002/ijc.30903

[48] Ritchie ME , Phipson B, Wu D, Hu Y, Law CW, Shi W, . limma powers differential expression analyses for RNA-sequencing and microarray studies. Nucleic Acids Res 2015;43:e47. 25605792 10.1093/nar/gkv007 PMC4402510

[49] Itraconazole . LiverTox: clinical and research information on drug-induced liver injury. Bethesda (MD): National Institute of Diabetes and Digestive and Kidney Diseases; 2012. 31643176

[50] Abdelmaseih R , Abdelmasih R, Hasan M, Tadepalli S, Patel J. Serious adverse events associated with hydroxychloroquine amidst COVID-19 pandemic: case series and literature review. Cureus 2020;12:e8415. 32626630 10.7759/cureus.8415 PMC7331778

[51] Boughton C , Taylor D, Ghataore L, Taylor N, Whitelaw BC. Mineralocorticoid hypertension and hypokalaemia induced by posaconazole. Endocrinol Diabetes Metab Case Rep 2018;2018:17–0157. 10.1530/EDM-17-0157 PMC5813713 29472988

[52] Pia A , Vignani F, Attard G, Tucci M, Bironzo P, Scagliotti G, . Strategies for managing ACTH dependent mineralocorticoid excess induced by abiraterone. Cancer Treat Rev 2013;39:966–73. 23582279 10.1016/j.ctrv.2013.03.003

