# Supplementary material for: A Phase I Study of Hydroxychloroquine and Suba-Itraconazole in Men with Biochemical Relapse of Prostate Cancer (HITMAN-PC): Dose Escalation Results
Source: Cancer Res Commun. 2026 Mar 27;6(3):687–97. doi: 10.1158/2767-9764.CRC-26-0010 (PMC13026449; doi:10.1158/2767-9764.CRC-26-0010)
Supplement: Supplementary Figure 1 — Representative images of six prostate cancer cell lines treated with Itraconazole alone or in combination with chloroquine (CQ) for 5 days. [file crc-26-0010_supplementary_figure_1_suppsf1.pptx]

## Slide 1
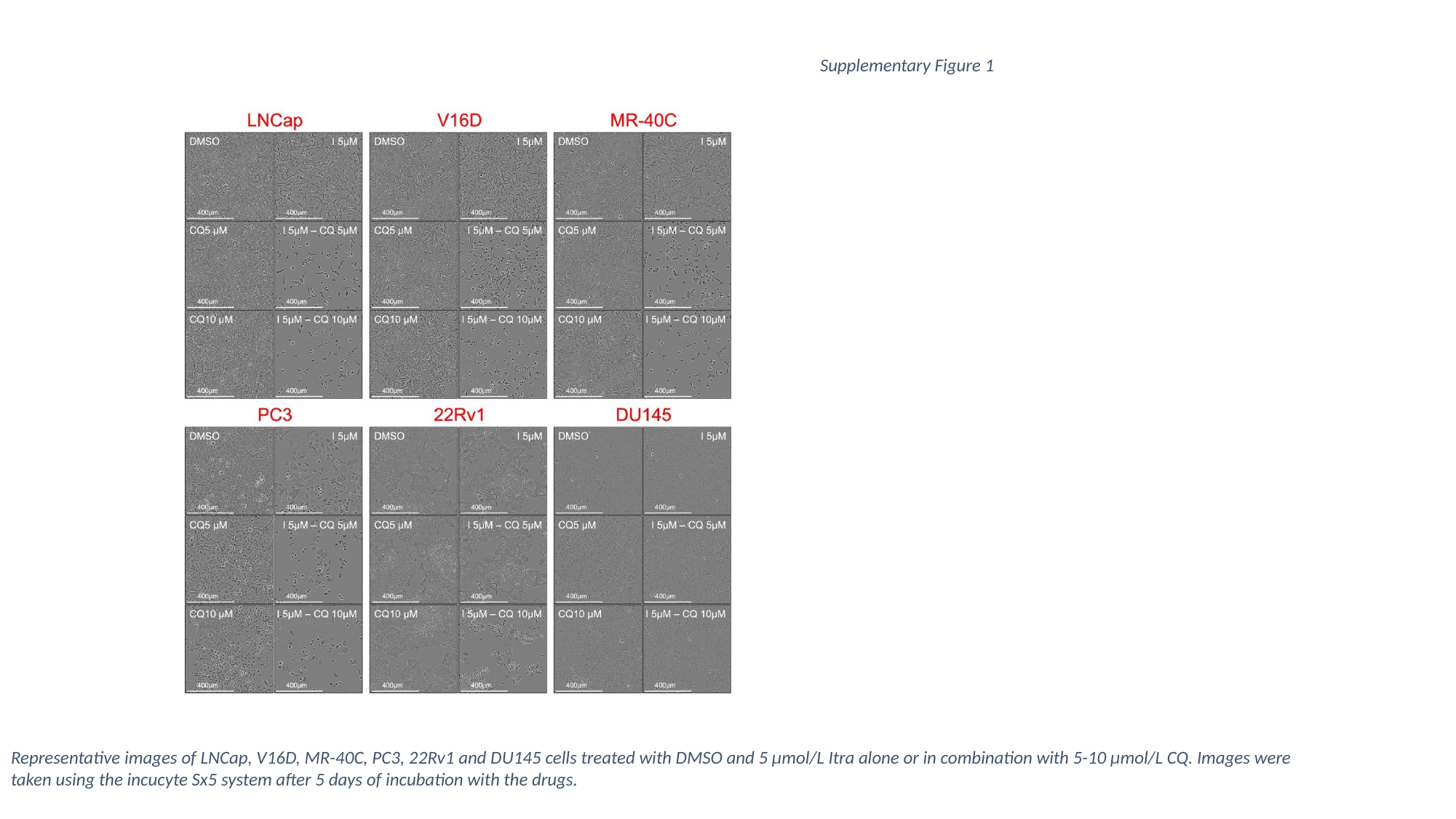

Supplementary Figure 1
Representative images of LNCap, V16D, MR-40C, PC3, 22Rv1 and DU145 cells treated with DMSO and 5 μmol/L Itra alone or in combination with 5-10 μmol/L CQ. Images were taken using the incucyte Sx5 system after 5 days of incubation with the drugs.
