# Supplementary material for: A Phase I Study of Hydroxychloroquine and Suba-Itraconazole in Men with Biochemical Relapse of Prostate Cancer (HITMAN-PC): Dose Escalation Results
Source: Cancer Res Commun. 2026 Mar 27;6(3):687–97. doi: 10.1158/2767-9764.CRC-26-0010 (PMC13026449; doi:10.1158/2767-9764.CRC-26-0010)
Supplement: Supplementary Figure 2 — Dose-response curves, 3D synergy maps, and histograms comparing the combined cytotoxic effect of Itraconazole with CQ or HCQ in LNCaP, V16D, and MR-40C cells. [file crc-26-0010_supplementary_figure_2_suppsf2.pptx]

## Slide 1
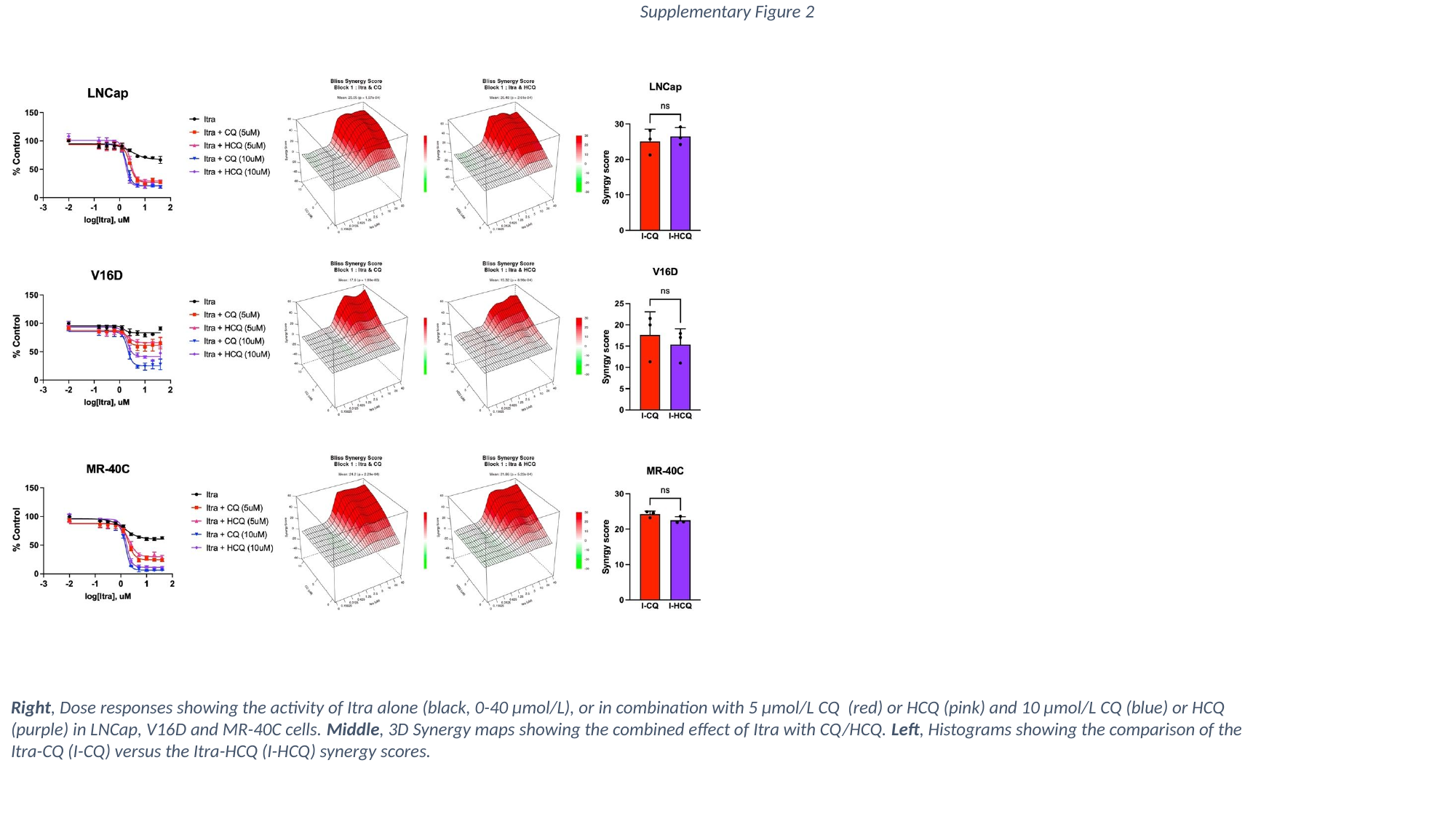

Supplementary Figure 2
Right, Dose responses showing the activity of Itra alone (black, 0-40 μmol/L), or in combination with 5 μmol/L CQ (red) or HCQ (pink) and 10 μmol/L CQ (blue) or HCQ (purple) in LNCap, V16D and MR-40C cells. Middle, 3D Synergy maps showing the combined effect of Itra with CQ/HCQ. Left, Histograms showing the comparison of the Itra-CQ (I-CQ) versus the Itra-HCQ (I-HCQ) synergy scores.
