# Supplementary material for: A Phase I Study of Hydroxychloroquine and Suba-Itraconazole in Men with Biochemical Relapse of Prostate Cancer (HITMAN-PC): Dose Escalation Results
Source: Cancer Res Commun. 2026 Mar 27;6(3):687–97. doi: 10.1158/2767-9764.CRC-26-0010 (PMC13026449; doi:10.1158/2767-9764.CRC-26-0010)
Supplement: Supplementary Figure 3 — Representative images of LNCaP, V16D, and MR-40C cells treated with Itraconazole alone or in combination with CQ or HCQ for 5 days [file crc-26-0010_supplementary_figure_3_suppsf3.pptx]

## Slide 1
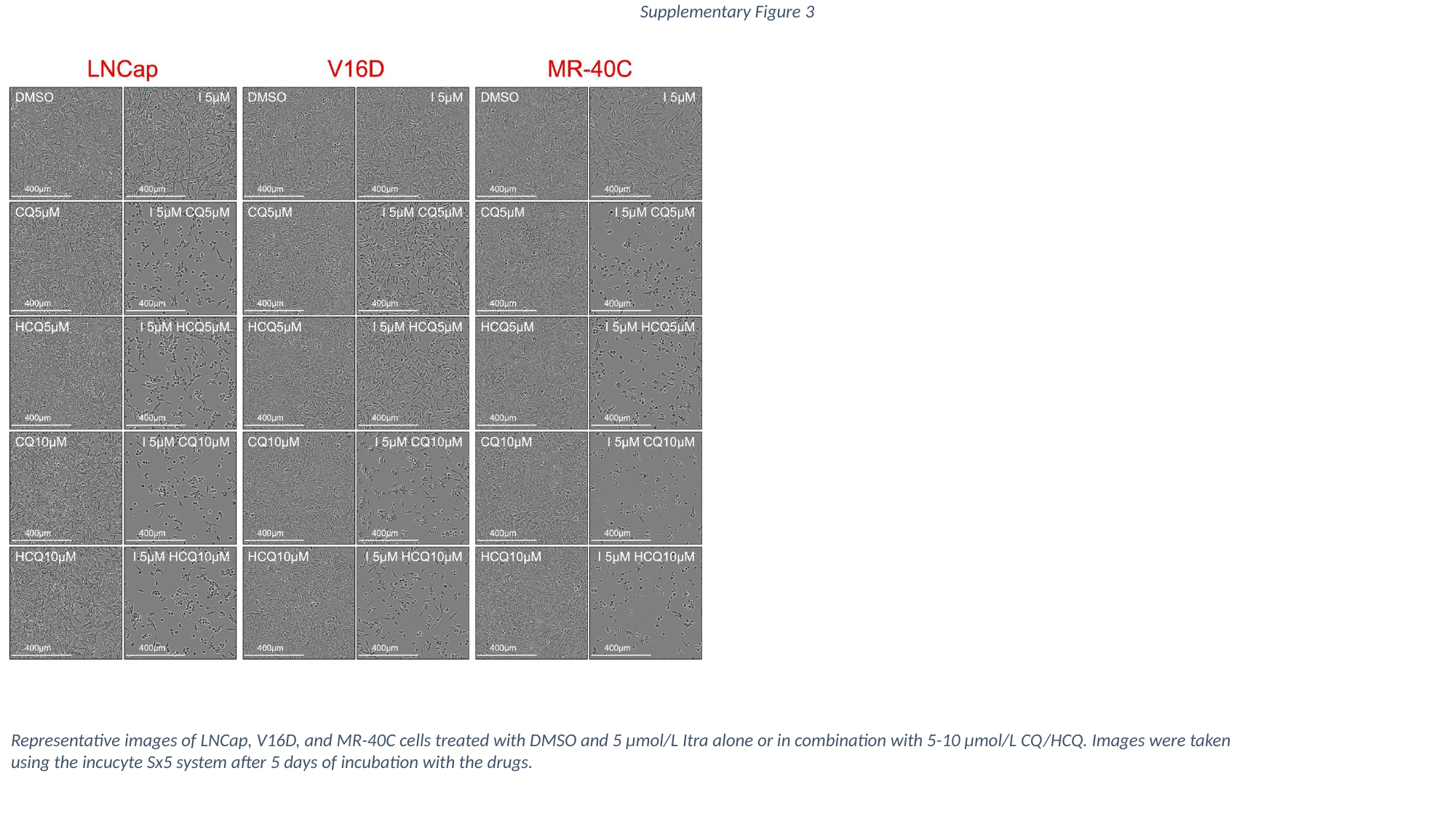

Supplementary Figure 3
Representative images of LNCap, V16D, and MR-40C cells treated with DMSO and 5 μmol/L Itra alone or in combination with 5-10 μmol/L CQ/HCQ. Images were taken using the incucyte Sx5 system after 5 days of incubation with the drugs.
