# Supplementary material for: A Phase I Study of Hydroxychloroquine and Suba-Itraconazole in Men with Biochemical Relapse of Prostate Cancer (HITMAN-PC): Dose Escalation Results
Source: Cancer Res Commun. 2026 Mar 27;6(3):687–97. doi: 10.1158/2767-9764.CRC-26-0010 (PMC13026449; doi:10.1158/2767-9764.CRC-26-0010)
Supplement: Supplementary Figure 4 — Heatmap showing the fold change of treatment-altered lipids that exhibited significantly different magnitudes of change across the three HCQ dose groups. [file crc-26-0010_supplementary_figure_4_suppsf4.pptx]

## Slide 1
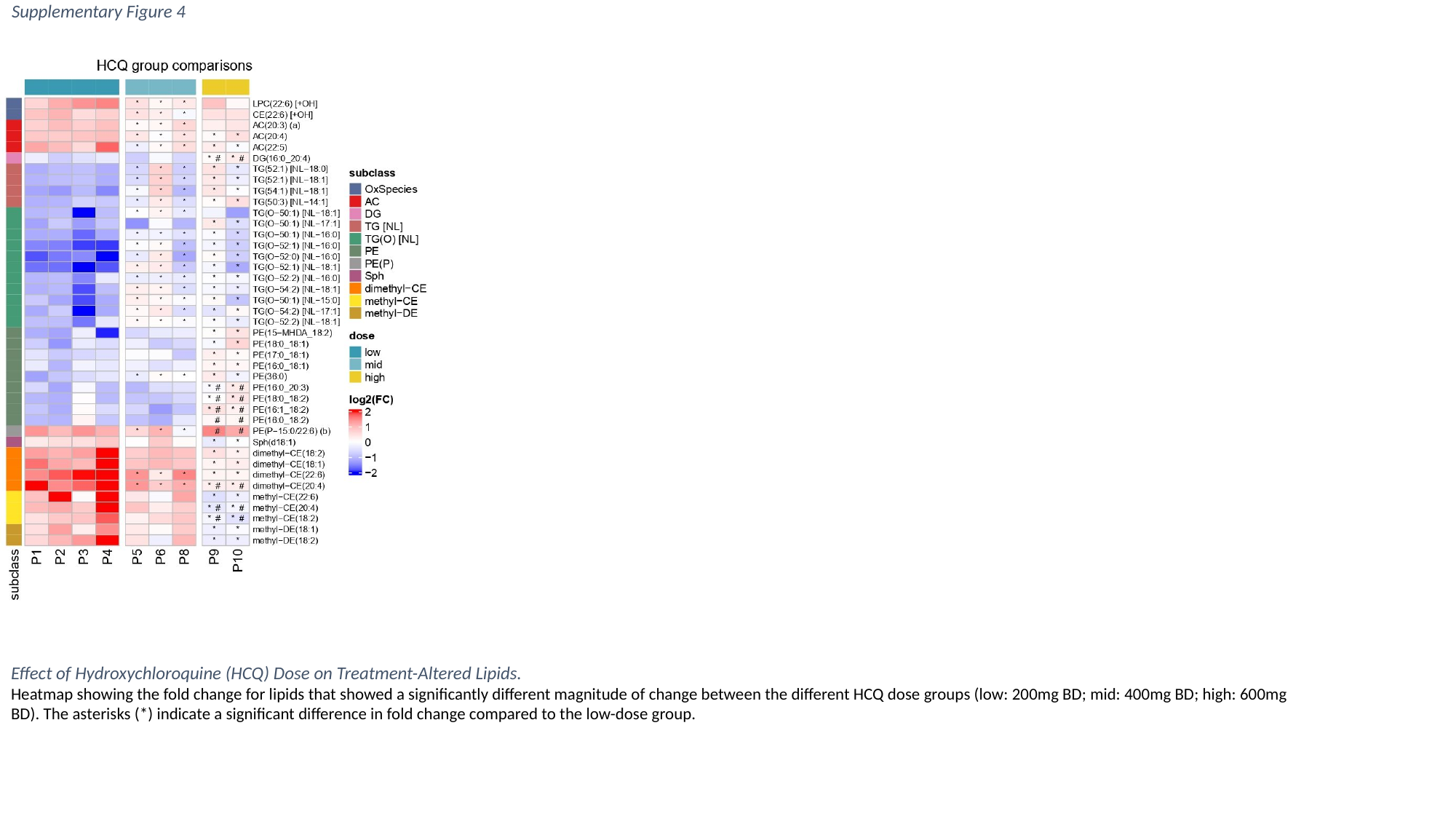

Supplementary Figure 4
Effect of Hydroxychloroquine (HCQ) Dose on Treatment-Altered Lipids.
Heatmap showing the fold change for lipids that showed a significantly different magnitude of change between the different HCQ dose groups (low: 200mg BD; mid: 400mg BD; high: 600mg BD). The asterisks (*) indicate a significant difference in fold change compared to the low-dose group.
