# Supplementary material for: A Phase I Study of Hydroxychloroquine and Suba-Itraconazole in Men with Biochemical Relapse of Prostate Cancer (HITMAN-PC): Dose Escalation Results
Source: Cancer Res Commun. 2026 Mar 27;6(3):687–97. doi: 10.1158/2767-9764.CRC-26-0010 (PMC13026449; doi:10.1158/2767-9764.CRC-26-0010)
Supplement: Supplementary Figure 5 — Scatter plots demonstrating significant correlations between the fold change of specific treatment-altered lipids (triacylglycerols, oxidized species, and sphingomyelins) and PSA-PFS time. [file crc-26-0010_supplementary_figure_5_suppsf5.pptx]

## Slide 1
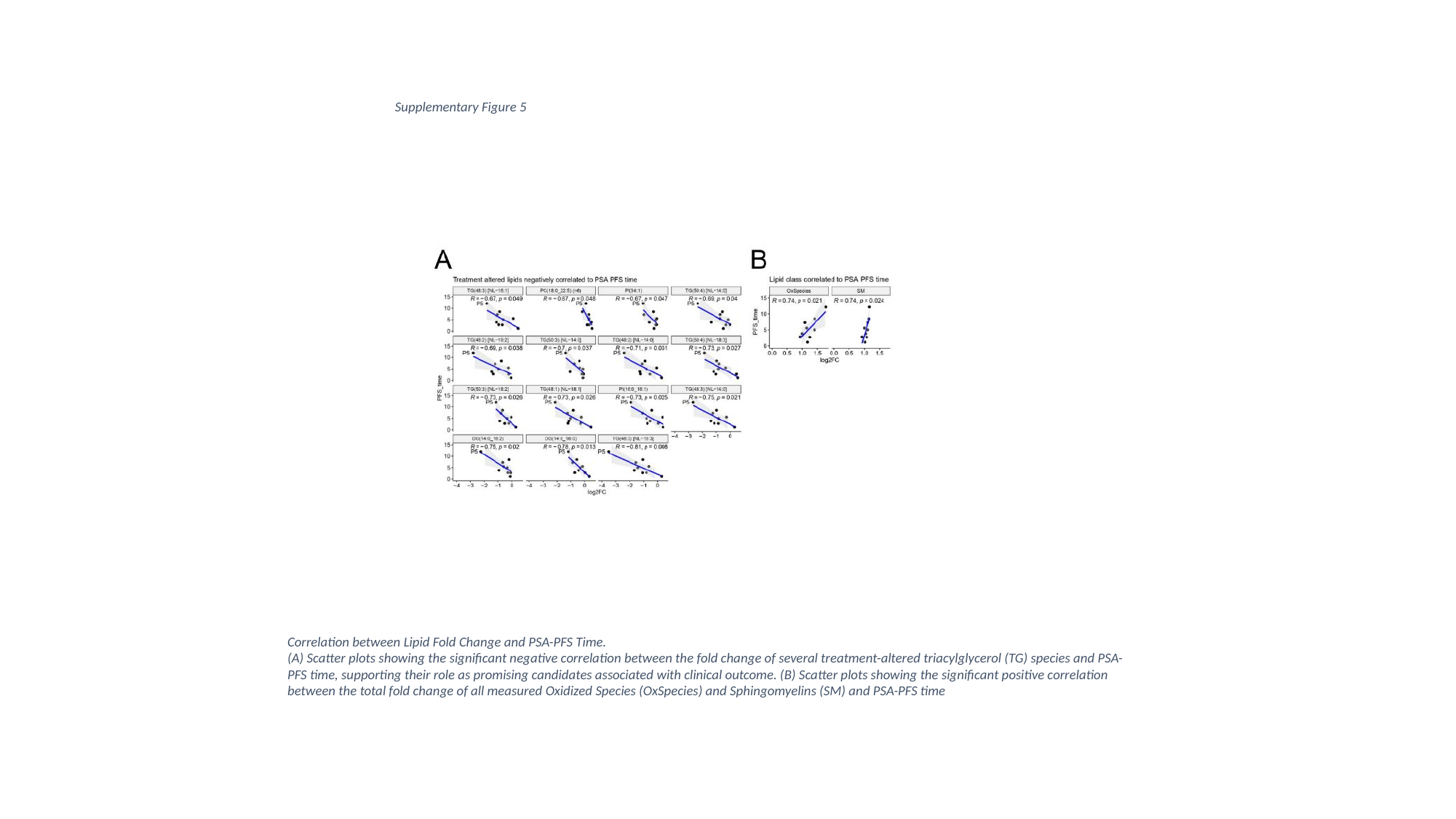

Supplementary Figure 5
Correlation between Lipid Fold Change and PSA-PFS Time.
(A) Scatter plots showing the significant negative correlation between the fold change of several treatment-altered triacylglycerol (TG) species and PSA-PFS time, supporting their role as promising candidates associated with clinical outcome. (B) Scatter plots showing the significant positive correlation between the total fold change of all measured Oxidized Species (OxSpecies) and Sphingomyelins (SM) and PSA-PFS time
