# Supplementary material for: A Phase I Study of Hydroxychloroquine and Suba-Itraconazole in Men with Biochemical Relapse of Prostate Cancer (HITMAN-PC): Dose Escalation Results
Source: Cancer Res Commun. 2026 Mar 27;6(3):687–97. doi: 10.1158/2767-9764.CRC-26-0010 (PMC13026449; doi:10.1158/2767-9764.CRC-26-0010)
Supplement: Supplementary Figure 6 — Heatmaps comparing plasma lipid fold changes between patient groups defined by alternative PSA-PFS time cutoffs. [file crc-26-0010_supplementary_figure_6_suppsf6.pptx]

## Slide 1
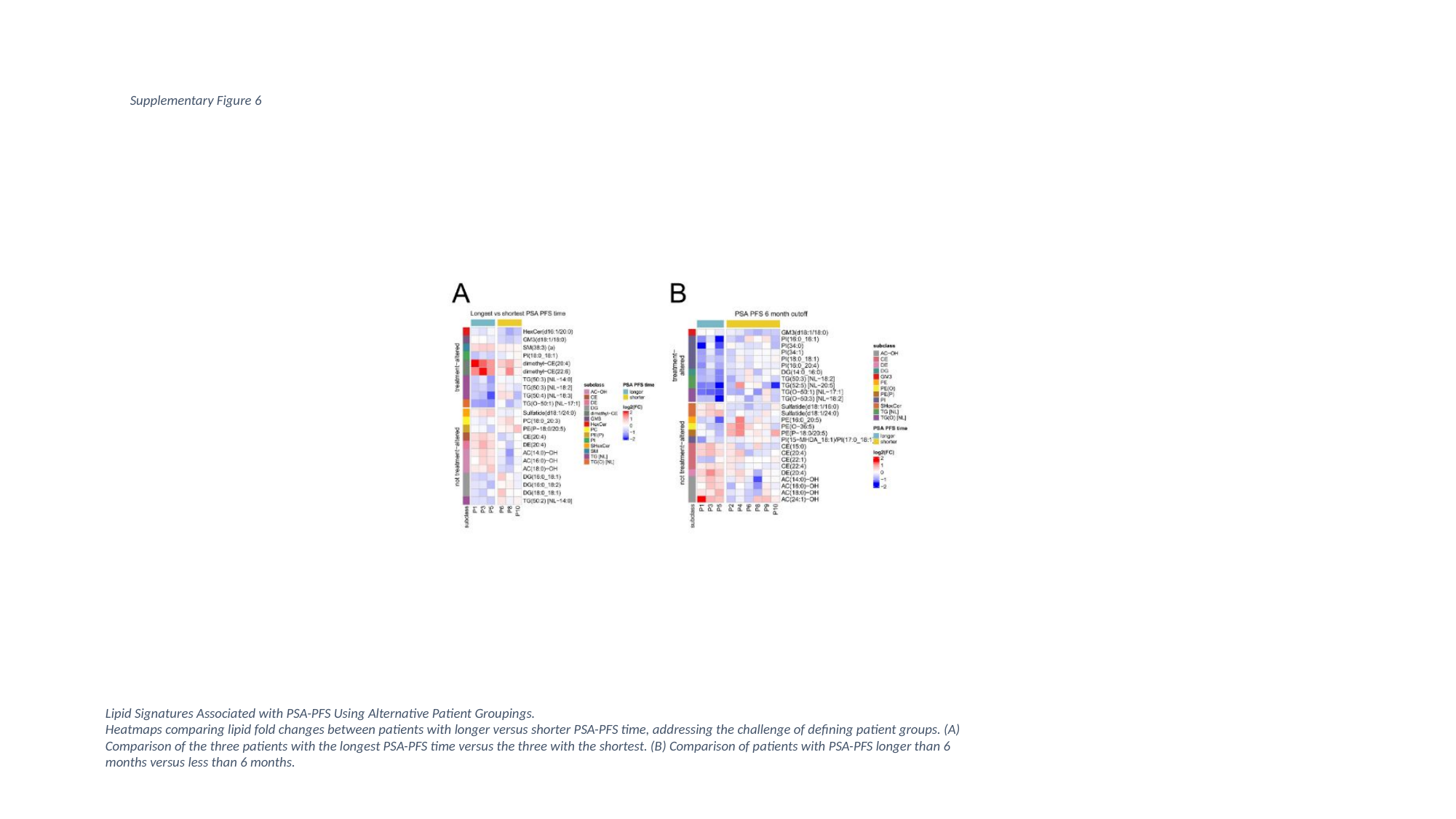

Supplementary Figure 6
Lipid Signatures Associated with PSA-PFS Using Alternative Patient Groupings.
Heatmaps comparing lipid fold changes between patients with longer versus shorter PSA-PFS time, addressing the challenge of defining patient groups. (A) Comparison of the three patients with the longest PSA-PFS time versus the three with the shortest. (B) Comparison of patients with PSA-PFS longer than 6 months versus less than 6 months.
