# Supplementary material for: A Phase I Study of Hydroxychloroquine and Suba-Itraconazole in Men with Biochemical Relapse of Prostate Cancer (HITMAN-PC): Dose Escalation Results
Source: Cancer Res Commun. 2026 Mar 27;6(3):687–97. doi: 10.1158/2767-9764.CRC-26-0010 (PMC13026449; doi:10.1158/2767-9764.CRC-26-0010)
Supplement: Supplementary Table 1 — Detailed enrichment analysis showing the differential lipid species and total lipid species altered post-treatment by sub-class. [file crc-26-0010_supplementary_table_1_suppst1.docx]

Supplementary Table 1. Representativeness of Study Participants

| **Considerations related to:** | **Biochemically Recurrent Prostate Cancer (BCR)** | **Overall representativeness of this study** |
| --- | --- | --- |
| **Sex** | Prostate cancer is an androgen-dependent malignancy occurring exclusively in men[1]. | The study enrolled 11 male participants (100%), consistent with the biology of the disease. |
| **Age** | The median age at prostate cancer diagnosis in Australia is 70 years[2] BCR typically occurs 20-38 months post-primary treatment[3]. | The median age of participants in this study was 73 years (range 69–77), which is representative of the older male population typically presenting with biochemical recurrence after prior definitive therapy. |
| **Race/Ethnicity** | Prostate cancer incidence and mortality rates vary by race. In Western populations, Black men often have higher incidence rates compared to White or Asian men[4]. In Australia (where the study was conducted), the majority of diagnosed men are Caucasian. | All participants (100%) were White. While this reflects the demographics of the specific referral center catchment area (St Vincent's Hospital, Sydney), it limits the assessment of racial differences in treatment response. |
| **Geography** | Prostate cancer is a global health burden. In Australia, it is the most commonly diagnosed cancer in men[2]. | The study was conducted at a single tertiary referral center in Sydney, Australia. Participants lived in the greater metropolitan area, which may limit generalizability to rural or non-urban populations. |

[1] Li C, Cheng D, Li P. Androgen receptor dynamics in prostate cancer: from disease progression to treatment resistance. Front Oncol 2025;15:1542811. https://doi.org/10.3389/fonc.2025.1542811.

[2] Cancer Council Australia. Prostate cancer 2024. https://www.cancer.org.au/cancer-information/types-of-cancer/prostate-cancer.

[3] Tourinho-Barbosa R, Srougi V, Nunes-Silva I, Baghdadi M, Rembeyo G, Eiffel SS, et al. Biochemical recurrence after radical prostatectomy: what does it mean? Int Braz J Urol 2018;44:14–21. https://doi.org/10.1590/S1677-5538.IBJU.2016.0656.

[4] Hansen M, Hamieh NM, Markt SC, Vaselkiv JB, Pernar CH, Gonzalez-Feliciano AG, et al. Racial Disparities in Prostate Cancer: Evaluation of Diet, Lifestyle, Family History, and Screening Patterns. Cancer Epidemiol Biomarkers Prev 2022;31:982–90. https://doi.org/10.1158/1055-9965.EPI-21-1064.
