# Supplementary material for: A Phase I Study of Hydroxychloroquine and Suba-Itraconazole in Men with Biochemical Relapse of Prostate Cancer (HITMAN-PC): Dose Escalation Results
Source: Cancer Res Commun. 2026 Mar 27;6(3):687–97. doi: 10.1158/2767-9764.CRC-26-0010 (PMC13026449; doi:10.1158/2767-9764.CRC-26-0010)
Supplement: Supplementary Table 2 — Comparison of the study participants' demographic characteristics with the general population of men with biochemically recurrent prostate cancer. [file crc-26-0010_supplementary_table_2_suppst2.docx]

**Supplementary Table 2:** Detailed Enrichment Analysis

| **Change** | **Lipid sub-class** | **Differential lipid species (% of sub-class)** | **Total lipid species in sub-class** | **Enrichment p-value** |
| --- | --- | --- | --- | --- |
| Decreased post-treatment | TG [NL] | 40 (52%) | 77 | <0.001 |
|  | TG(O) [NL] | 16 (80%) | 20 | <0.001 |
|  | PE | 19 (52%) | 37 | <0.001 |
|  | HexCer | 9 (64%) | 14 | <0.001 |
| Increased post-treatment | AC | 14 (50%) | 28 | <0.001 |
|  | LPC(O) | 7 (70%) | 10 | <0.001 |
|  | Cer(m) | 7 (64%) | 11 | <0.001 |
|  | dimethyl-CE | 4 (100%) | 4 | <0.001 |
|  | OxSpecies | 6 (67%) | 9 | <0.001 |
|  | LPC | 19 (31%) | 61 | 0.001 |
|  | deDE | 3 (100%) | 3 | 0.004 |
|  | Sph | 3 (100%) | 3 | 0.004 |
|  | PC(O) | 8 (36%) | 22 | 0.014 |
|  | methyl-DE | 2 (100%) | 2 | 0.025 |
|  | methyl-CE | 3 (60%) | 5 | 0.030 |
